# Mental Health Functioning in the Human Rights Field: Findings from an International Internet-Based Survey

**DOI:** 10.1371/journal.pone.0145188

**Published:** 2015-12-23

**Authors:** Amy Joscelyne, Sarah Knuckey, Margaret L. Satterthwaite, Richard A. Bryant, Meng Li, Meng Qian, Adam D. Brown

**Affiliations:** 1 Department of Psychiatry, Bellevue Hospital Center, New York University School of Medicine, Program for Survivors of Torture, New York, New York, United States of America; 2 Human Rights Institute, Columbia Law School, New York, New York, United States of America; 3 Center for Human Rights and Global Justice, New York University School of Law New York, New York, United States of America; 4 School of Psychology, University of New South Wales, Sydney, New South Wales, Australia; 5 Department of Psychiatry, Steven and Alexandra Cohen Veterans Center, New York University School of Medicine, New York, New York, United States of America; 6 Department of Psychology, Sarah Lawrence College, Bronxville, New York, United States of America; University of Stellenbosch, SOUTH AFRICA

## Abstract

Human rights advocates play a critical role in promoting respect for human rights world-wide, and engage in a broad range of strategies, including documentation of rights violations, monitoring, press work and report-writing, advocacy, and litigation. However, little is known about the impact of human rights work on the mental health of human rights advocates. This study examined the mental health profile of human rights advocates and risk factors associated with their psychological functioning. 346 individuals currently or previously working in the field of human rights completed an internet-based survey regarding trauma exposure, depression, posttraumatic stress disorder (PTSD), resilience and occupational burnout. PTSD was measured with the Posttraumatic Stress Disorder Checklist-Civilian Version (PCL-C) and depression was measured with the Patient History Questionnaire-9 (PHQ-9). These findings revealed that among human rights advocates that completed the survey, 19.4% met criteria for PTSD, 18.8% met criteria for subthreshold PTSD, and 14.7% met criteria for depression. Multiple linear regressions revealed that after controlling for symptoms of depression, PTSD symptom severity was predicted by human rights-related trauma exposure, perfectionism and negative self-appraisals about human rights work. In addition, after controlling for symptoms of PTSD, depressive symptoms were predicted by perfectionism and lower levels of self-efficacy. Survey responses also suggested high levels of resilience: 43% of responders reported minimal symptoms of PTSD. Although survey responses suggest that many human rights workers are resilient, they also suggest that human rights work is associated with elevated rates of PTSD and depression. The field of human rights would benefit from further empirical research, as well as additional education and training programs in the workplace about enhancing resilience in the context of human rights work.

## Introduction

Human rights advocates investigate alleged violations of human rights around the world and advocate for the cessation, prevention, and remedy of abuses. The human rights field has grown significantly over the past several decades, with thousands of groups identifying themselves as human rights organizations. Human rights work often involves direct or indirect exposure to events that may be traumatic, including through interviewing witnesses and victims of abuse, reviewing documentation and evidence of harm (e.g. forensic reports, videos of assaults), visiting physical sites of abuse (e.g. prisons, mass graves), witnessing violations, and being located in insecure environments. It is well documented in numerous contexts that exposure to trauma may have adverse impacts on mental health. However, little attention has been paid in mental health research to the psychological functioning of human rights advocates specifically. Similarly, within the field of human rights itself, responses to issues such as vicarious trauma are often inadequate, with little in-depth discussion and few resources available. In an effort to begin to address these gaps, we conducted a cross-sectional survey on-line to examine mental health issues in human rights advocates, as well as occupational and psychological variables that might be associated with mental health outcomes.

To date, there is limited research about potential negative mental health impacts of human rights work. Holtz and colleagues [1] showed that 70 human rights advocates in Kosovo were at risk for mental health issues as a significant minority of participants reported elevated levels of anxiety (17.1%), depression (8.6%), and posttraumatic stress disorder (PTSD; 7.1%). Additionally, duration of human rights work, exposure to direct threats, and working in a hostile environment predicted negative mental health outcomes. Despite these findings linking human rights work with poor mental health in Kosovo, studies have yet to examine whether these types and magnitude of psychological issues are found across a wider range of human rights contexts and little remains known about the factors that predict negative or positive outcomes in this population. Support for the hypothesis that human rights advocates may be at risk for negative mental health impacts may be deduced from research on other professions whose work and trauma exposure overlaps with or is similar to human rights advocates, including humanitarian workers [2] police [3], rescue workers [4], journalists [5] and veterans [6]. Studies on these other populations indicate that the risk of developing mental health issues in the wake of trauma is associated with a number of factors including, but not limited to, the type and duration of exposure to the traumatic event, an individual’s prior history of trauma exposure, and perceived lack of mental health training and emotional support within the workplace (for reviews see [2,7]). Clarifying those factors that might place human rights advocates at risk for mental health issues is needed, as the level and type of exposure to trauma can vary considerably in this field. Human rights work, for example, can include a myriad of occupational tasks, ranging from work that involves direct or indirect exposure to potentially traumatic events or material such as witnessing violence and human rights violations, reviewing physical and forensic evidence, interviewing or providing legal advice to traumatized individuals, to tasks that may not include trauma exposure, such as data analysis and political lobbying. In addition, human rights advocates sometimes become victims themselves of violence and human rights violations due to the nature or location of their work.

In addition to occupation-related variables, a number of psychological factors may impact mental health issues in human rights advocates. Existing studies from other settings show that a strong predictor of negative mental health outcomes following adversity is the extent to which people believe they have been permanently changed by the event and make catastrophic negative self-appraisals about themselves and their world [8–9]. Another factor known to contribute to poor adaptation to stress and trauma is perfectionism, which involves persistent striving for flawlessness with self-criticism of one’s own behavior [10]. Furthermore, increasing evidence points to perceptions of agency or control over aversive situations as a strong predictor of positive adaptation [11].

Accordingly, we hypothesized that negative mental health impacts in human rights advocates would be predicted by a history of childhood trauma, occupational factors such as greater trauma exposure, and individual factors such as negative self-appraisals, perfectionism, and low self-efficacy.

## Methods

### Participants

Participants were recruited through networks of human rights advocates and organizations. The survey was disseminated through contacts with a broad swathe of human rights organizations and professional networks. A recruitment notice was sent to a variety of international human rights organizations, including Amnesty International, Global Rights, Human Rights First, Human Rights Watch, and Physicians for Human Rights. The notice was also sent to regional and national human rights organizations in many countries, as well as specialized organizations focusing on specific rights or communities. In several instances, senior officials at the organizations sent the recruitment notice to their staff, and in others, research and program staff circulated the notice on internal email lists after being approached through professional connections. A variety of list-serves circulated the recruitment notice, including lists comprising women's human rights defenders, LGBTQ advocates, and directors of international human rights law clinics. The notice was also sent to staff at the UN Office of the High Commissioner for Human Rights in Geneva and in field offices in a variety of countries. Twitter and FaceBook were also used to disseminate the recruitment notice.

The survey was available online for completion from October, 2012 to February, 2013.

Participants provided informed consent and confirmed that they were at least 18 years old and had experience in the human rights field in order to participate. We obtained written informed consent from participants before the study began. The study was approved by the New York University’s University Committee on Activities Involving Human Subjects.

### Measures

#### Demographics

Participants were asked to indicate their age, gender, nationality, and number of years working in the field of human rights. In addition, participants were asked to indicate on a scale of 1–4 (1 = None, 2 = Minimal, 3 = Moderate, 4 = A Lot) the extent to which they carried out various human rights duties over the course of their career.

#### Trauma-exposure

Human rights work-related trauma exposure was assessed through questions concerning trauma exposure through (e.g. listening to accounts of violations, witnessing human rights abuses, viewing graphic videos or photos, threats or completed acts of being taken hostage, sexual assault, detention). Participants were asked to indicate if they had been exposed to each of these events and then to estimate on a scale of 1–4 (1 = None, 2 = Minimal, 3 = Moderate, 4 = A Lot) the extent to which they were exposed to each of these events throughout their work in human rights.

In addition, individuals were asked if they had been exposed to non-human rights related work before or after the age of 18. If they indicated trauma exposure they were asked to estimate the number of times these events occurred before and after the age of 18.

#### PTSD

PTSD symptoms were measured using the PTSD Checklist-Civilian Version (PCL-C) [12]. The PCL-C is a 17-item self-report scale based on DSM-IV PTSD criteria that evaluates how much participants have experienced PTSD symptoms in the past month as a result of stressful life events. Each item is scored on a 5-point scale (1 = “not at all,” 5 = “extremely”). A diagnosis of PTSD was determined when an individual met DSM-IV symptom criterion defined by the presence of at least 1 B item (questions 1–5), 3 C items (questions 6–12), and at least 2 D items (questions 13–17). Symptoms rated as "Moderately" or above (responses 3 through 5 on individual items) were counted as present. In addition to this distribution of symptoms, the total symptom severity score had to be 44 or greater. Individuals were determined to meet sub-threshold PTSD when they met criteria for two of the three symptom clusters.

#### Depression

Depressive symptoms were measured using the Patient Health Questionnaire (PHQ-9) [13]. The measure consists of 9 items, each rated on a 4-point scale (0 = “not at all,” 3 = “nearly every day”) in the past two weeks, resulting in total scores that range from 0–27. The total PHQ-9 score was calculated by summing the responses for each symptom. A cut-off score of 10 was used as an indicator of probable major depression.

#### Resilience

Resilience was operationalized as the absence of persistent psychological distress, despite exposure to trauma (as distinct from the absence of mental disorder) [14]. To be consistent with previous studies that have identified trajectories of resilience following trauma exposure as having minimal PTSD symptoms, resilience was operationalized as a PCL score of ≤ 25 (e.g. [15–16]).

#### Burnout

Burnout was defined as “taking at least three months off from work due to psychological distress or burnout.” Individuals were asked to provide a yes/no response to this single item question.

#### Centrality of Human Rights Work

Centrality of human rights work (“Centrality”) refers to the extent to which a person views their human rights work as central to his or her self-identity and life story. Centrality was measured with a modified version of the seven-item Centrality of Event Scale (CES-70) [8]. For the purposes of the present study, instead of asking about a specific traumatic event, participants rated on a 5-point scale (1 = “totally disagree,” 5 = “totally agree”) how central their experiences in human rights work are to their self-identity (e.g. “working in the field of human rights permanently changed my life”).

#### Perfectionism

To index trait perfectionism, we administered the “unrelenting” standards subscale of the Young Schema Questionnaire (YSQ) [17]. These eight items index the extent to which respondents make excessive demands upon themselves (e.g. “My health is suffering because I put myself under so much pressure to do well”) on a six-item scale (1 = “completely untrue of me,” 6 = “describes me perfectly”).

#### Self-efficacy

Self-efficacy was measured using the General Self-Efficacy (GSE) scale [18], which assesses personal agency and perceived ability to cope with challenges that one encounters in life. The scale consists of 10 statements with a 4-point scale (1 = “not at all true,” 4 = “exactly true”).

#### Human Rights-Related Self-Appraisals

In light of the importance of appraisal style in determining how people respond to trauma, a series of statements about working in human rights were constructed based on discussions with professionals in the field. These statements included sentences with both positive and negative valences. The positive scale included items such as “I have been able to make a positive difference through my work,” and “I feel inspired by my work,” and the negative appraisal scale included items such as “I feel that my work is pointless.” The positive human rights-related items had a Cronbach’s alpha of 0.85, while the Cronbach’s alpha for the negative human rights-related appraisals was 0.78. A full list of items is available from the authors upon request.

### Statistical Analysis

SPSS was used to conduct the prevalence analyses and correlations. SAS 9.4 and R version 3.0.3 were used to conduct the simple and multiple regressions. Pearson correlations were conducted between PTSD and occupational responsibilities.

In order to examine the associative factors with PTSD and Depression, the PCL-C and PHQ-9 scores were transformed to approximately normal distributions. Since PCL-C scores were all positive and PHQ-9 scores were non-negative values including zeros, the Box-Cox transformation method [19] was used to transform PCL-C, while the Yeo-Johnson transformation [20] method which permits zero responses was used to transform PHQ-9. The transformed scores were then scaled by multiplying 100. The one-parameter Box–Cox transformation is defined as:

$$y_{i}^{\left(\lambda \right)}=\left\{ \begin{array}{c} \frac{y_{i}^{\lambda }-1}{\lambda }\;if\;\lambda \neq 0,\\ \text{ln}(y_{i})\;if\;\lambda =0. \end{array}\right.$$ (1)

The one-parameter Yeo-Johnson transformation for non-negative responses is defined as:

$$y_{i}^{\left(\lambda \right)}=\left\{ \begin{array}{l} \;\frac{{\left(y_{i}+1\right)}^{\lambda }-1}{\lambda }\;if\;\lambda \neq 0,y_{i}\geq 0\\ \text{ln}(y_{i}+1)\;if\;\lambda =0,y_{i}\geq 0 \end{array}\right.$$ (2)

The transformation parameter λ in formula (1) was selected as -0.54 for PCL-C and parameter λ in formula (2) was selected as 0.16 for PHQ-9 based on the maximum log-likelihoods. A series of simple linear regressions were conducted to show the bivariate relationship between explanatory variables and outcome variables (PCL-C and PHQ-9). Those variables that were significantly associated with the PCL-C and PHQ-9 in the simple linear regression analyses were then entered as predictor variables into the multiple linear regression models to test their effects on predicting the PCL-C and PHQ-9, controlling for possible confounders. All tests of significance were two-tailed with significance level of .05.

Occupational predictors of burnout were examined using multiple logistic regression.

## Results

### Demographics

In total, 524 participants began the survey and 346 participants completed all or almost all measures, resulting in a completion rate of 66%. The remainder of the analyses in this paper focus on the 346 completers, comprising 256 females and 75 males, with a mean age 37.73 years (*SD* = 12.01), representing individuals of 51 nationalities.

### Prevalence of Exposure

Human rights advocates who responded to the survey were exposed to a wide range of potentially traumatic events in their work. A large percentage (34.4% [*n* = 119] - 89.3% [*n* = 309]) of survey respondents directly witnessed trauma towards others or were indirectly exposed to trauma through work with clients, survivors, and witnesses (see Table 1). In addition, a significant minority of human rights advocates (6.4% [*n* = 22]– 20.5% [*n* = 71]) were themselves victims of violence or direct threats, such as being threatened or actually being taken hostage, assaulted, or arrested (see Table 1). Furthermore, a considerable percentage of survey respondents were exposed to non-human right work-related trauma, such as natural disasters, motor vehicle accidents and physical and sexual assault; importantly, these exposures occurred both prior to (3.8% [*n* = 13] - 20.8% [*n* = 72]) and following 18 years of age (6.9% [*n* = 24] - 32.7% [*n* = 113], see Table 2).

**Table 1:** Frequency and type of trauma exposure reported in Human Rights Advocates.

Exposure Type	Frequency
Conducting HR Interviews	89.3.%
Visiting Sites of Violations	63.3%
Witnessed Violence	34.4%
Witnessed Violations of Basic Needs	78.9.%
Responding to Emergency	54.6%
Taken Hostage, Beaten, Assaulted	6.4%
Threatened with Hostage, Beaten, or Assault	20.5%
Arrested by a Government	20.2%

**Table 2:** Frequency and type of non-human rights work trauma exposure.

Non-HRW Exposure	Before 18	After 18
Natural Disaster	14.5%	24.6%
Accident (e.g. MVA)	19.9%	32.7%
Physical or Sexual Assault	20.8%	21.7%
Combat	3.8%	6.9%

MVA = Motor Vehicle Accident. HRW = Human Rights Work.

### Prevalence of Distress

The mean PCL-C score was 31.6 (*SD* = 12.9), with 19.4% (*n* = 67), of individuals meeting full *DSM-IV* criteria for PTSD, and 18.8% (*n* = 65) meeting criteria for subthreshold PTSD (two out of the three symptom clusters). The mean score on the PHQ-9 was 5.4 (*SD* = 5.0), with 14.7% (*n* = 55) of individuals scoring in the range of probable major depression.

Based on our operationalization of Resilience (PCL-C score of ≤ 25), 42.5% (*n* = 134), of respondents indicated that they were resilient despite the nature of their work. When we categorized these respondents according to exposure to severe trauma exposure, defined as an event in which a person was directly threatened or attacked, however, 28.4% (*n* = 33) of the trauma-exposed group was resilient.

### Occupational Correlates of Distress

Ten human rights work-related duties representing a range of activities with differing likelihoods of trauma exposure (monitoring, litigation, lobbying, education, quantitative analysis, aid or medical care, handling evidence, conducting interviews, visiting sites, working with human rights victims) were correlated with PTSD symptom severity (see Table 3). Interestingly, although these occupational tasks ranged considerably, all but two of the tasks were positively associated with PTSD severity. Only engagement in litigation and providing direct aid and medical care to human rights victims were not associated with PTSD.

**Table 3:** Correlations between occupational task and symptoms of PTSD.

		1	2	3	4	5	6	7	8	9	10	11
1.	PCL-C	1										
2.	Monitoring	.27^**^	1									
3.	Litigation	.07	.20^**^	1								
4.	Lobbying	.12^*^	.25^**^	.11^*^	1							
5.	Education	.13^*^	.16^**^	.27^**^	.31^**^	1						
6.	Quantitative analysis	.19^**^	.14^*^	.01	.17^**^	.18^**^	1					
7.	Aid or medical care	.05	.09	-.05	.02	.18^**^	.08	1				
8.	Handling evidence	.25^**^	.29^**^	.33^**^	.17^**^	.13^*^	.21^**^	-.04	1			
9.	Conducting interviews	.22^**^	.35^**^	.33^**^	.15^**^	.17^**^	.10	.06	.48^**^	1		
10.	Visiting sites	.30^**^	.47^**^	.16^*^	.18^**^	.15^*^	.23^**^	.09	.50^**^	.48^**^	1	
11.	Working with victims	.31^**^	.34^**^	.08	.19^**^	.08	.16^**^	.17^**^	.30^**^	.27^**^	.50^**^	1

PCL-C = PTSD Checklist-Civilian Version.

*p < .05,

** *p* < .01.

### Associative Factors of PTSD and Depression

Separate linear regressions were conducted to examine the factors that contribute to PTSD and depression. First, a Kolmogorov–Smirnov test [21] determined that the distributions of the PCL-C and the PHQ-9 were not normally distributed (PCL-C: K–S = .13, *p* < .01; PHQ-9: K–S = .15, *p* < .01). Next, the PCL-C and PHQ-9 scores were transformed using the Box-Cox transformation method [19] and the Yeo-Johnson transformation method [20] respectively to meet the assumptions of the linear regression models. A series of simple linear regression models were then conducted using each variable of PHQ-9 (or PCL-C), age, number of years in human rights work, number of traumatic events experienced in human rights work, number of traumatic events experienced prior to the age of 18, positive self-appraisals, negative self-appraisals, perfectionism, self-efficacy, and centrality as the single predictor, and using the PCL-C (or PHQ-9) as the outcome variable (See Tables 4 and 5). PTSD symptom severity was positively associated with symptoms of depression (*p* < .0001), human rights-related trauma exposure (*p* < .0001), trauma exposure before age 18 (*p* < .05), perfectionism (*p* < .0001), centrality (*p* < .0001) and negative appraisals (*p* < .0001). Depression symptom severity was positively associated with PTSD symptom severity (*p* < .0001), human rights-related trauma exposure (*p* < .01), trauma exposure before age 18 (*p* < .01), perfectionism (*p* < .0001), negative self-appraisals (*p* < .0001), centrality (*p* < .01) as well as, negatively associated with self-efficacy (*p* < .01) and age (*p* < .05). Those variables that were significantly associated with the PCL-C and the PHQ-9 were then entered as predictor variables into two separate multiple linear regressions.

**Table 4:** Simple linear regression models predicting PTSD.

	Estimated Coefficient	Standard Error	*t*	*p*
Depression	0.028	0.003	10.450	< .0001
Age	-0.050	0.027	-1.828	0.069
Years in HR	0.313	0.255	1.229	0.220
HR Exposure	1.228	0.207	5.939	< .0001
Exposure <18	0.717	0.358	2.005	0.046
Pos.Appraisals	0.032	0.043	0.745	0.457
Neg.Appraisals	0.370	0.039	9.411	< .0001
Self-Efficacy	-0.159	0.145	-1.093	0.275
Perfectionism	0.341	0.047	7.284	< .0001
Centrality	0.282	0.062	4.582	< .0001

Depression = Patient Health Questionnaire-9 (PHQ-9). HR Exposure = Human Rights-related Trauma Exposure. Exposure < 18 = Trauma Exposure before age 18. Self-Efficacy = General Self Efficacy Scale (GSE). Perfectionism = Young Schema Questionnaire (YSQ). Centrality = Centrality of Human Rights Work.

**Table 5:** Simple linear regression models predicting depression.

	Estimated Coefficient	Standard Error	*T*	*p*
PTSD	8.868	0.849	10.450	< .0001
Age	-1.162	0.491	-2.367	0.019
Years in HR	-3.247	4.626	-0.702	0.483
HR Exposure	11.251	3.887	2.894	0.004
Exposure <18	22.649	6.533	3.467	0.001
Pos.Appraisals	-1.350	0.766	-1.763	0.079
Neg.Appraisals	4.986	0.758	6.577	< .0001
Self-Efficacy	-7.923	2.541	-3.118	0.002
Perfectionism	5.970	0.824	7.244	< .0001
Centrality	3.693	1.111	3.325	0.001

*Notes*. PTSD = Posttraumtic Stress Disorder Checklist-Civilian Version (PCL-C). HR Exposure = Human Rights-related Trauma Exposure. Exposure < 18 = Trauma Exposure before Age 18. Self-Efficacy = General Self Efficacy Scale (GSE). Perfectionism = Young Schema Questionnaire (YSQ). Centrality = Centrality of Human Rights Work.

Result summaries (estimated coefficients, standard errors, t values and p values) of the multiple linear regression model predicting PTSD are presented in Table 6. Human rights-related trauma exposure, depressive symptoms, perfectionism, and negative self-appraisals about human rights work have significant effects on predicting PTSD symptom severity in the model.

**Table 6:** Multiple linear regression analysis predicting PTSD.

	Estimated Coefficient	Standard Error	*T*	*p*
HR Exposure	0.872	0.195	4.476	< .0001
Depression	0.016	0.003	5.382	< .0001
Exposure<18	-0.380	0.339	-1.120	0.264
Perfectionism	0.126	0.049	2.588	0.010
Neg. Appraisals	0.249	0.040	6.185	< .0001
Centrality	0.092	0.057	1.629	0.105

*Notes*. HR Exposure = Human Rights-related Trauma Exposure. Exposure < 18 = Trauma Exposure before Age 18. Perfectionism = Young Schema Questionnaire (YSQ). Centrality = Centrality of Human Rights Work.

Result summaries (estimated coefficients, standard errors, t values and p values) of the multiple linear regression model predicting depression are presented in Table 7. PTSD symptoms, perfectionism, and lower levels of self-efficacy have significant effects on predicting depressive symptoms in the model.

**Table 7:** Multiple linear regression analysis predicting depression.

	Estimated Coefficient	Standard Error	*t*	*p*
HR Exposure	-3.984	4.194	-0.950	0.343
Exposure<18	11.940	6.779	1.761	0.079
PTSD	6.500	1.196	5.436	< .0001
Perfectionism	3.075	0.974	3.158	0.002
Age	-0.406	0.497	-0.817	0.415
Neg. Appraisals	0.485	0.900	0.539	0.590
Self-Efficacy	-7.046	2.622	-2.687	0.008
Centrality	1.246	1.183	1.053	0.293

*Notes*. HR Exposure = Human Rights-related Trauma Exposure. Exposure < 18 = Trauma Exposure before Age 18. PTSD = Posttraumatic Stress Disorder Checklist-Civilian Version (PCL-C). Perfectionism = Young Schema Questionnaire (YSQ). Self-Efficacy = General Self Efficacy (GSE). Centrality = Centrality of Human Rights Work.

### Occupational Factors Associated with Burnout

Nineteen percent (*n* = 67) of survey responders indicated that they have taken a break of three months or more from HR work at some stage due to burnout. A binary logistic regression was conducted examining the extent to which different types of human rights work predicts burnout. Four types of human rights work were included in the model based on the authors’ expectations about the likelihood of trauma exposure associated with different kinds human-rights work, “low threat of trauma exposure” (e.g. secondary data analysis, survey development, education), “involving secondary trauma exposure” (e.g. conducting interviews, reviewing evidence, working with traumatized clients), “witnessing trauma” (e.g. direct exposure through witnessing human rights violations”), and “personal threat or experience of trauma” (e.g. being threatened with or actual exposure to violence or detention in the context of human rights work). Overall, the model was significant (*x* ^2^ = 16.30, *df* = 4, *p* < .01) indicating that the predictors reliably distinguished between individuals who did and did not take time off of human rights work due to psychological distress. Among the four predictor variables in the model, “involving secondary trauma exposure” (*B* = .52, *p* < .05, *OR =* 1.68, lower limit: 1.05, upper limit: 2.68) and “witnessing trauma” (*B* = .29, *p* < .05, *OR =* 1.33, lower limit: .54, upper limit: 1.28) predicted burnout.

## Discussion

Consistent with previous findings about people exposed to significant levels of trauma and adversity [22], we observed that a large percentage of those human rights workers who responded to the survey appeared resilient in terms of their psychological functioning. Despite this overall pattern of coping with human rights work, we observed elevated rates of psychopathology. These rates indicate that human rights advocates may be at significant risk of PTSD and depression.

These findings begin to shed light on those factors that are associated with poorer mental health among human rights advocates. Human rights-related trauma exposure, perfectionism, and negative human rights-related self-appraisals predicted PTSD symptom severity, whereas depression symptoms were predicted by perfectionism and lower levels of self-efficacy.

It has often been reported that greater trauma exposure predicts PTSD [23]. The predictive role of negative self-appraisals is expected as other studies have shown that negative self-appraisals about one’s self or exaggerated perceptions of vulnerability to future harm predict the onset and maintenance of PTSD [23]. Interestingly, because we adapted our questions to target appraisals that were relevant to human rights, the finding that negative self-appraisals was a strong predictor of PTSD underscores that the interpretation that workers place on their experiences and themselves is pivotal in terms of how they manage the psychological effects of their work. Although these findings show a strong link between negative self-appraisals with PTSD, future work would benefit from considering the role of other kinds of appraisals (e.g., performance reviews) as a predictor of psychological well-being.

The finding that perfectionism contributed to poor mental health is consistent with other studies showing that it predicts burnout [24]. It appears that overly high expectations of oneself as a human rights advocate, despite the limited capacity to influence all situations, places the worker at increased risk. This finding is consistent with evidence that self-blame predicts negative mental health outcomes in the wake of trauma [25]. Having excessively high expectations about one’s ability to meaningfully impact the people and situations entailed in human rights work may compound the stress of this work, and seems to be one individual difference that renders some advocates more at risk of mental health difficulties.

Only two types of human rights work were not associated with PTSD–engagement in litigation, and providing direct aid and medical care to victims. Further research is required to understand this finding, which might be connected to different occupational structures for action in the face of abuse, or perceptions of concrete accomplishment or direct assistance to specific individuals, which might provide a buffer against PTSD [26,27]. However, future research would be needed to examine the relation between the role of varied occupational circumstances and mental health outcomes in the human rights advocate population.

Impaired self-efficacy predicted depression. This finding is consistent with previous work showing a link between self-efficacy and better management of adversity in general,^11^ as well as studies showing that higher levels of self-efficacy are associated with lower levels of depression in trauma exposed populations [28,29]. In the context of human rights work, it is possible that an internal belief that one is not able to adequately control or impact outcomes either for oneself or for victims of human rights abuses contributes to a sense of helplessness, which in turn contributes to depression.

A number of limitations in this study need to be taken into account. First, the study consisted of a self-selected convenience sample, thereby introducing unknowable biases into sampling. We used online data collection, not clinical interviews, although there is some evidence that online versus face-to-face assessment can function comparably [30]. The rates of PTSD and depression may be inflated because of the self-report format, especially in that the PTSD measure did not index the impairment criterion required to make a PTSD diagnosis. Second, the study was cross-sectional in nature and future studies employing a prospective design will allow for a better understanding of the mental health predictors and outcomes to human rights work. Third, estimates of trauma exposure within the context of human rights work is difficult to determine as survey responders might have also been exposed to traumatic events in the same setting as the human rights work, but not directly associated with their job. Thus, future studies would benefit from clarifying the rates of trauma exposure in relation to the human rights work along with exposure that occurs outside of the work. Along those lines, future studies would benefit from examining the geographic scope of where human rights advocates work in relation to mental health outcomes in this population. Finally, resilience in this study was operationalized as a score < 25 on the PCL-C. This index was based on longitudinal studies examining trajectories of mental health symptoms following exposure to trauma. The validity of using this index in a cross-sectional design warrants further research.

Despite the study’s limitations, the findings have implications for the human rights field. First, the prevalence of PTSD and depression points to the need for organizations and supervisors to ensure the adequate provision of mental health monitoring and evidence-based interventions. Second, these findings also point to potential opportunities for educating and preparing human rights advocates for the mental health risks associated with their work. It may be useful to train human rights advocates to understand the signs and symptoms of psychopathology, as well as strategies for developing realistic self-appraisals, reducing perfectionism, and developing ways to enhance self-efficacy. Finally, despite the magnitude of psychological distress observed in our study, a large proportion of human rights advocates demonstrated resilience. Further research to understand the factors associated with resilience in this population is needed and could provide an evidence base for interventions aimed not only at preventing and reducing morbidity but also at enhancing resilience and career longevity. Overall, these findings highlight the psychological costs of the stressors endured by human rights advocates, and the need for additional research to better prepare, support, and sustain human rights advocates in their work.
